# Advancements in MitraClip Intervention for Mitral Regurgitation: A Comprehensive Review and Comparative Analysis of Clinical Trials

**DOI:** 10.7759/cureus.54805

**Published:** 2024-02-24

**Authors:** Shitij Shrivastava, Shashwat Shrivastava, Sai Vishnu Vardhan Allu, Patrik Schmidt, Moiud Mohyeldin, Abeer Qasim

**Affiliations:** 1 Medicine, BronxCare Health System, New York, USA; 2 Cardiothoracic Surgery, New York University, New York, USA; 3 Internal Medicine, BronxCare Health System, New York, USA

**Keywords:** cardiothoracic surgery, mitral valve regurgitation, mitraclip, tanscatheter mitral valve repair, cardiology devices

## Abstract

This comprehensive review explores the evolution and clinical impact of MitraClip intervention in the management of mitral regurgitation. Mitral regurgitation results from dysfunction in the mitral valve (MV) apparatus. The MitraClip Clip Delivery System was approved by the Food and Drug Administration (FDA) in 2013. The discussion delves into the procedural foundation of MitraClip intervention, primarily based on Alfieri’s technique of edge-to-edge leaflet approximation. As highlighted by key clinical trials, including Endovascular Valve Edge-to-Edge Repair (EVEREST) II Trial, Cardiovascular Outcomes Assessment of the MitraClip Percutaneous Therapy for Heart Failure Patients with Functional Mitral Regurgitation (COAPT) Trial, and Percutaneous Repair with the MitraClip Device for Severe Functional/Secondary Mitral Regurgitation (MITRA-FR) trial, the efficacy and safety of MitraClip were evaluated in comparison to surgical interventions and guideline-directed medical therapy. Notably, the COAPT demonstrated significant benefits in reducing all-cause mortality and heart failure hospitalization, while the MITRA-FR presented contrasting results, emphasizing the importance of patient selection. An analysis of the EVEREST II trial underscores MitraClip's potential to achieve comparable outcomes to surgical intervention, emphasizing its role in reducing mitral regurgitation and improving clinical status. However, limitations and complications, such as device-related issues and the potential impact on future MV surgery, are discussed. The study also explores the evolving landscape of MV interventions, reflecting advancements and the growing acceptance of MitraClip. In conclusion, the MitraClip device represents a significant advancement in the treatment of mitral regurgitation. The data presented highlights its promising results in terms of reduced hospitalization rates, improved in-hospital mortality, and enhanced quality of life for patients. However, challenges remain, and careful consideration of patient selection and underlying pathology is crucial in determining the optimal treatment approach. Ongoing research and clinical experience will continue to refine our understanding of MitraClip's role in the evolving landscape of MV interventions.

## Introduction and background

The management of valvular heart diseases has evolved significantly in recent years, with transcatheter interventions emerging as a viable alternative to traditional surgical approaches. Conditions such as severe aortic stenosis and mitral regurgitation present complex challenges, and advancements in transcatheter techniques, exemplified by MitraClip and transcatheter aortic valve replacement, have revolutionized treatment options. This paradigm shift is underscored by studies exploring outcomes, complications, and comparative effectiveness, influencing clinical decision-making. As the field continues to develop, a comprehensive understanding of the background and current state of transcatheter interventions for valvular heart diseases is crucial for informed and effective patient care. The mitral valve (MV) apparatus consists of the mitral annulus, valve leaflets, chordae tendineae, and papillary muscles [1]. Mitral regurgitation occurs when these components dysfunction, allowing blood to flow back into the left atrium during systole. Mitral regurgitation is a prevalent valvular condition, affecting up to 10% of the general population [2]. Treatment options include medications, surgery (replacement or repair), and transcatheter edge-to-edge repair (TEER). MitraClip (Clip Delivery System) was approved by the Food and Drug Administration (FDA) in 2013 for percutaneous MV repair in patients with prohibitive surgical risk and degenerative MV regurgitation. Interventional cardiology has made dramatic progress in the field of percutaneous transcatheter-based treatment of valvular heart disease in recent years [3]. In this traditional review, we will review the literature regarding MitraClip percutaneous transcatheter MV repair as a treatment option for secondary mitral regurgitation.

## Review

Transcatheter MVr is principally based on Alfieri’s technique of edge-to-edge approximation of anterior and posterior mitral leaflets in the area of maximum leak, creating a double-orifice MV to reduce regurgitation [4]. This only deals at the leaflet level to curb regurgitation to acceptable limits and multiple clips can be used in the areas of leakage while carefully monitoring the gradients [5]. Since its FDA approval in 2013, its application has expanded considerably [6].

In the data published by Selena et al. in August 2021, trends of MV intervention for mitral valvular regurgitation were analyzed from 2000 to 2016. The International Classification of Diseases, Ninth Revision (ICD-9) or International Classification of Diseases, Tenth Revision (ICD-10) procedure codes were used to identify the patients older than 18 years of age who underwent mitral valve replacement (MVR), MVr, or MitraClip implantation. Selena et al. studied a total of 656,032 patients undergoing mitral interventions from 2000 to 2016, excluding patients with mitral stenosis and infective endocarditis. The group mean age was significantly higher in the MitraClip group as compared to the MVR or MVr groups (77.0 vs. 64.7 vs. 64.4 years) (p < 0.001). The majority of patients in the MVR group were females, whereas males were the predominant gender in the MVr and MitraClip cases. The predominant race was White in all three subsets. Procedures were mostly elective. However, MitraClip patients were sicker than the MVR or MVr groups with a mean Elixhauser Comorbidity Score (ECS) of 10.1, 6.8, and 6.2 (p < 0.001), respectively.

Promising results were observed in the average length of stay (LOS) and in-hospital mortality for all interventions. The average median LOS in days per year reduced to 0.85 (p = 0.03) (2013-2016) in the MitraClip group over the specified period of time. The overall LOS lowered significantly for all interventions from 8.8 days to 7.0 days. The in-house mortality rate also decreased tremendously in the MitraClip group from 3.6% to 1.5% (2013-2016). The overall mortality rate dropped from 8.5% to 3.7%. Reduction in LOS and in-hospital mortality may be attributed to improved technology and better post-intervention care expediting the discharge process. After the FDA approval for MitraClip in 2013, its usage leaped to 10.2% in 2016 from 1.0% in 2013, bestowing a significant growth rate of 84.4% annually (p < 0.001). A considerable number of patients belonged to greater surgical risk who otherwise would have undergone MVr, thus keeping the total number of mitral interventions the same. This was a huge acceptance of MitraClip in dealing with patients with severe MR with congestive cardiac failure. Comparatively, MVR experienced a significant dip of 5.6% annually (p < 0.001) from 2000 to 2010, while the MVr rate increased to 8.4% per year (p < 0.001) from 2000 to 2006 and later both procedures stabilized. However, with concomitant coronary artery bypass grafting, the rate of MVR increased proportionally in 2008 [7].

Endovascular Valve Edge-to-Edge Repair II (EVEREST II) Trial

In 2011, Ted and colleagues published data of the Endovascular Valve Edge-to-Edge Repair II (EVEREST II) Trial where they randomized 279 patients in 2:1 distribution (September 2005 to November 2008) having moderately severe (grade 3+) or severe (grade 4+) mitral regurgitation to undergo either transcatheter percutaneous repair or MV surgery. They included 37 centers in the United States and Canada. The criteria for symptomatic patients were a left ventricular ejection fraction (LVEF) of more than 25% and a left ventricle end-systolic diameter of 55 mm or less. The inclusion criteria for asymptomatic patients were LVEF between 25% and 60%, left ventricle end-systolic diameter of 40 to 55 mm, and new-onset atrial fibrillation or pulmonary hypertension [8,9]. The anatomical inclusion criteria were MR jet primarily from non-coaptation of anterior and posterior leaflets [9,10].

The primary composite endpoint for efficacy was freedom from all-cause death, surgery for MV dysfunction, and grade 3+ or more mitral regurgitation at one year. The primary safety endpoint was the rate of major adverse events at 30 days, defined as the composite of death, myocardial infarction, emergent cardiac surgery, bacteremia, gastrointestinal complication requiring surgery, renal failure, stroke, deep wound infection, mechanical ventilation for more than 48 hours, new-onset permanent atrial fibrillation, transfusion of at least two units of blood, and redo surgery for failed MV surgery. The secondary endpoints were changes in the LV dimension and volumes, New York Heart Association (NYHA) functional class [9,11], and quality of life scores [9,12]. Out of 279 patients, 178 underwent transcatheter intervention and 80 patients underwent MV surgery. Twenty-one patients withdrew their consent for either procedure. A portion (23%) of the patients in the percutaneous group developed 3+ to 4+ MR and were referred for surgery. The rate of primary efficacy endpoints achieved were 55% and 73% in the transcatheter and surgery groups, respectively (p = 0.007). At two years, the rate of surgery for MV dysfunction in the percutaneous repair group vs. surgery group was 22% vs. 4%, and the rates of surgery for 3+ or 4+ MR were 20% vs. 22%. Overall, the primary endpoint was observed in 52% of the patients in the percutaneous repair group vs. 66% of patients in the surgery subgroup at 24 months (p = 0.04). The rate of major adverse events was 48% in patients who underwent surgery and 15% in patients who underwent percutaneous intervention at one month (p < 0.001). Secondary endpoints fared better in the percutaneous subsets at 12 and 24 months and at the end of 12 months [9]. 

Cardiovascular Outcomes Assessment of the MitraClip Percutaneous Therapy for Heart Failure Patients With Functional Mitral Regurgitation (COAPT) Trial

The Cardiovascular Outcomes Assessment of the MitraClip Percutaneous Therapy for Heart Failure Patients with Functional Mitral Regurgitation (COAPT) Trial data were published in 2018, where the researchers studied outcomes in symptomatic patients with heart failure (HF) and grade 3+ or 4+ MR who underwent TEER. These patients were symptomatic despite being on maximally tolerated guideline-directed medical therapy (GDMT). Secondary MR is defined as an effective regurgitant orifice area (EROA) >30 mm^2^ and/or regurgitant volume >45 mL and LVEF ≥20% [13].

A total of 614 patients were randomized with 302 patients in the MitraClip + GDMT group and 312 patients in the GDMT group. Device implantation was embarked upon 96% of those patients. More than half (63%) of the patients were male, while the mean age was 72 years. The patients were followed up for two years. Certain inclusion and exclusion criteria were laid down for the study. These patients were symptomatic despite maximally tolerated GDMT, had an ejection fraction between 20% and 50%, and had grade 3+ or grade 4+ MR. Mitral stenosis, life expectancy of less than one year, and concomitant cardiac procedures were some of the exclusion criteria. Abbott sponsored the trial. The primary endpoint was divided into the primary effectiveness endpoint and primary safety endpoint. The primary effectiveness endpoint was hospitalization for HF from any cause within 24 months of follow-up, and the primary safety endpoint was freedom from device-associated complications at 12 months post-procedure. Multiple secondary endpoints were established as discussed below [13].

The primary effectiveness endpoint was observed in 35.8% and 67.9% for the MitraClip + GDMT and GDMT alone groups, respectively, at 24 months (p < 0.001). The primary safety endpoint was achieved in 96.6% of the patients (p < 0.001). With respect to the secondary endpoints, all-cause mortality was 29.1% vs. 46.1% (95% confidence interval (CI) 0.46-0.82; p < 0.001) in the device and control groups, respectively. The mean change in the LV end-diastolic volume at 12 months compared to baseline was -3.7 ml vs. 17.1 ml (p = 0.004) in the device and control groups, respectively. In two years, the decrease in the severity of MR by ≤2+ compared to baseline was 99.1% vs. 43.4% (p < 0.001) in the MitraClip + GDMT vs. GDMT groups. Although not significant, at two years, the rates of death or hospitalization due to HF in NYHA class IV patients were lower in the device subgroup compared to GDMT alone (66.7% vs. 85.2%; p: 0.86). A similar primary endpoint was observed among the patients less than 74 years (37.3% vs. 64.5%) and ≥74 years (51.7% vs. 69.6%) old. There was no significant interaction between age (<74 vs. ≥74 years) and randomized treatment with regard to the primary endpoint (p = 0.10). These findings suggest that patients who underwent transcather-based therapy had a statistically significant lower hospitalization for HF, all-cause mortality at 24 months, and therefore better outcomes. The quality of life was much improved in the device subgroup compared to the GDMT subgroup, which was measured via a standard questionnaire (scores were 12.5 ± 1.8 vs. -3.6 ± 1.9 in the device vs. control groups; p < 0.001) and six-minute walk test [13]. 

Percutaneous Repair With the MitraClip Device for Severe Functional/Secondary Mitral Regurgitation (MITRA-FR) Trial

The COAPT Trial did show that patients with secondary MR who underwent TEER had a lower rate of hospitalization from HF exacerbation and all-cause mortality compared to patients who only received medical therapy. However, contradicting results were published in the Percutaneous Repair with the MitraClip Device for Severe Functional/Secondary Mitral Regurgitation (MITRA-FR) Trial. It was sponsored by Abbott and the French Ministry of Health and Research National Program. This trial was conducted exclusively in France [14]. In this randomized controlled phase III trial, 304 patients with severe symptomatic HF and secondary mitral regurgitation were randomly assigned in a 1:1 ratio to undergo either transcatheter percutaneous MVr in addition to receiving guideline-directed medical therapy or guideline-directed medical therapy alone. Secondary MR was defined as an EROA >20 mm^2^ and/or a regurgitant volume >30 mL and LVEF between 15% and 40%. The MitraClip device by Abbott Vascular was used. The primary endpoint was all-cause mortality and hospitalization for HF at 12 months after randomization. Multiple secondary endpoints were established. These included death from cardiovascular causes, individual components of primary endpoint, freedom from major adverse cardiovascular events (including death, stroke, unplanned hospitalization, and myocardial infarction), NYHA class, MR severity, change in LV end-diastolic and systolic volumes, change in EF, brain natriuretic peptide levels, and functional assessment. Patients had similar baseline characteristics (with the exception of a higher history of myocardial infarction (49.3% vs. 34.2%) and diabetes (32.9% vs. 25.7%) in the device group) and were on similar GDMT. Technical success with the Abbott Mitraclip device was observed in 95.8% of patients who had the device implanted. The composite primary outcome of death from any cause or unintended hospitalization at 12 months was 54.6% in the intervention group and 51.3% in the control group (p = 0.53). Diving into it further, 24.3% of deaths occurred in the device group and 22.4% in the control group. The p-value was not statistically significant. In the intervention group, 48.7% of the patients had unintended hospitalization for HF vs. 47.4% in the GDMT alone group. A large amount of data on the secondary endpoints was missing, so it will not be discussed. The authors came to the conclusion that TEER is not superior to medical therapy when it comes to managing HF with reduced ejection fraction with secondary MR [14,15]. Based on the findings from COAPT, TEER has gained an extended approval from the FDA in the United States. 

There were many similarities between the two trials, but the COAPT trial enrolled more than double the number of patients and was conducted in almost quadruple the number of centers. According to a recent comparative literature [15], patients in MITRA-FR had significantly higher left ventricular end-diastolic volume (MITRA-FR: 135 ± 35 mL/m^2^ vs. COAPT: 101 ± 34 mL/m^2^), indicating substantially progressed LV disease. MITRA-FR did not randomize patients based on LV diameter, while COAPT excluded patients with severe LV dysfunction (LV end-systolic diameter <70 mm). Patients in the MITRA-FR group had less severe MR (EROA: 31 ± 10 mm²) relative to COAPT (41 ± 15 mm²). Hence, LV dysfunction was the primary etiology of MR and HF in MITRA-FR, while secondary MR was more likely to be the primary valvular pathology in COAPT. Patients with severe LV dysfunction may not benefit from TEER [14,15]. In the COAPT trial, the research protocol involved an examination of all HF medications taken by participants before their enrollment, with adjustments made to reach the highest tolerable doses. By contrast, MITRA-FR did not implement a similar protocol.

Effect of MitraClip implantation on future MV surgery

After the MitraClip implantation procedure, leaflets may get damaged, inhibiting the chances of repair. In this study [16], after MitraClip intervention, 41% of the patients underwent replacement in whom repair was planned pre procedure, 51% underwent repair as planned at onset, 5% underwent replacement as planned before, and 3% underwent repair in whom replacement was planned. Valve injury was noticed in 37% of the patients after MitraClip implantation. The data also reported similar 12-month MVr rates after the MitraClip procedure or de novo surgery, i.e., 89% vs. 84% (p = 0.36). This trial concludes that MitraClip implantation is the most appropriate initial procedure in patients who are at high surgical risk for MVr [16]. Table 1 provides a summary of the articles discussed.

**Table 1:** Summary of the articles discussed EVEREST II: Endovascular Valve Edge-to-Edge Repair, COAPT: Cardiovascular Outcomes Assessment of the MitraClip Percutaneous Therapy for Heart Failure Patients with Functional Mitral Regurgitation, MITRA-FR: Percutaneous Repair with the MitraClip Device for Severe Functional/Secondary Mitral Regurgitation, MV: mitral valve, GDMT: guideline-directed medical therapy, LV: left ventricular

Study/trial	Year	Intervention/Treatment	Inference
EVEREST II [9,10]	2011	MitraClip vs. mitral valve surgery	Primary efficacy endpoint achieved in 55% (MitraClip) vs. 73% (surgery) [9]. Significant MR reduction in both groups at 24 months [9].
COAPT [13]	2018	MitraClip + GDMT vs. GDMT alone	Primary effectiveness endpoint: 35.8% (MitraClip + GDMT) vs. 67.9% (GDMT alone) at 24 months [13]. Improved quality of life in MitraClip subgroup [13].
MITRA-FR [14,15]	2018	MitraClip + GDMT vs. GDMT alone	No significant difference in all-cause mortality and hospitalization at 12 months [14]. Higher LV volume in MITRA-FR patients compared to COAPT [15].
MitraClip impact on MV surgery [16]		MitraClip implantation	Leaflet damage post MitraClip placement: 41% replacement, 51% repair [16]. Valve injury in 37% of patients after MitraClip implantation [16].

The treatment landscape for mitral regurgitation is evolving rapidly, and MitraClip has emerged as a valuable tool in the armamentarium of interventions. However, careful patient selection, consideration of the underlying pathology, and long-term implications on surgical options must be taken into account when determining the most appropriate treatment approach for individual patients.

Limitations and complications of the MitraClip Clip Delivery System

The MitraClip Clip Delivery System is a safe and established procedure in today’s landscape. Nonetheless, it comes with a variety of complications and has its own limitations of use. They may be cardiac, vascular, renal, device-related, or procedure-related. Cardiac complications include pericardial effusion, iatrogenic atrial septal defect (iASD), and rarely endocarditis and atrial fibrillation. Pericardial effusion is rare, pertaining to safer transseptal puncture and delivery systems [17]. iASD has been reported in 24% of patients in one year. Those patients had a larger atrial diameter and more severe tricuspid regurgitation at baseline [18]. The incidence of de novo atrial fibrillation is reported to be 2.4% [19]. Device-related complications include persistent mitral regurgitation, mitral stenosis, clip embolization, single leaflet device attachment (SLDA), and injury to the mitral valvular apparatus. EVEREST II reported MR reduction ≤2+ in 77% of patients [9,17]. Operator experience and procedural planning are the factors that help minimize residual MR [17,20]. The MV orifice area ≤4.0 cm² is an important predictor of mitral stenosis after MitraClip implantation and is associated with poor outcomes [21]. Clip embolization can happen and clips have been reported being found in the right axillary artery in one case [22] and stuck to the LV apex in another [23].

## Conclusions

The management of mitral regurgitation has witnessed significant advancements in recent years, particularly in the realm of transcatheter interventions. The MitraClip device, approved by the FDA in 2013, has emerged as a promising option for patients with severe mitral regurgitation, especially those at high surgical risk. The data presented here highlight the evolution of MV interventions over time, with MitraClip gaining acceptance and being utilized in an increasing number of cases. Notably, MitraClip has shown promising results in terms of reduced hospitalization rates, improved in-hospital mortality, and enhanced quality of life for patients. The EVEREST II trial provided valuable insights into the efficacy of MitraClip compared to surgical intervention, demonstrating its potential to yield comparable outcomes in terms of reducing mitral regurgitation and improving clinical status. However, the landscape of clinical trials also presents challenges, as seen in the contrasting outcomes of the COAPT and MITRA-FR trials. While COAPT demonstrated the benefits of MitraClip in reducing all-cause mortality and hospitalization for heart failure, MITRA-FR did not find a significant difference between transcatheter intervention and medical therapy alone. These discrepancies underscore the importance of patient selection and the underlying etiology of mitral regurgitation in determining the efficacy of MitraClip. Furthermore, the impact of MitraClip implantation on the possibility of future MV surgery is a critical consideration. The potential for leaflet damage and the need for replacement rather than repair after MitraClip intervention have been highlighted. Further research and clinical experience will continue to refine our understanding of the role of MitraClip in the management of mitral regurgitation.
